# Treg and Oligoclonal Expansion of Terminal Effector CD8^+^ T Cell as Key Players in Multiple Myeloma

**DOI:** 10.3389/fimmu.2021.620596

**Published:** 2021-02-23

**Authors:** Douglas E. Joshua, Slavica Vuckovic, James Favaloro, Ka Hei Aleks Lau, Shihong Yang, Christian E. Bryant, John Gibson, Phoebe Joy Ho

**Affiliations:** ^1^ Institute of Haematology, NSW Health Pathology, Royal Prince Alfred Hospital, Camperdown, NSW, Australia; ^2^ Faculty of Medicine and Health, The University of Sydney, Sydney, NSW, Australia; ^3^ School of Life Sciences, University of Technology Sydney, Ultimo, NSW, Australia

**Keywords:** T cells, myeloma, oligoclonal expansions, CD69^+^T_TE_, CD39^-^Treg

## Abstract

The classical paradigm of host-tumor interaction, i.e. elimination, equilibrium, and escape (EEE), is reflected in the clinical behavior of myeloma which progresses from the premalignant condition, Monoclonal Gammopathy of Unknown Significance (MGUS). Despite the role of other immune cells, CD4^+^ regulatory T cells (Treg) and cytotoxic CD8^+^ T cells have emerged as the dominant effectors of host control of the myeloma clone. Progression from MGUS to myeloma is associated with alterations in Tregs and terminal effector CD8^+^ T cells (T_TE_). These changes involve CD39 and CD69 expression, affecting the adenosine pathway and residency in the bone marrow (BM) microenvironment, together with oligoclonal expansion within CD8^+^ T_TE_ cells. In this mini-review article, in the context of earlier data, we summarize our recent understanding of Treg involvement in the adenosine pathway, the significance of oligoclonal expansion within CD8^+^ T_TE_ cells and BM-residency of CD8^+^ T_TE_ cells in MGUS and newly diagnosed multiple myeloma patients.

## Introduction 

Multiple Myeloma (MM) is a malignancy of plasma cells which grow predominantly in the bone marrow (BM). The classical paradigm of host tumor interaction, i.e. elimination, equilibrium, and escape (EEE) is reflected in the clinical behavior of myeloma. The clinical counterparts of equilibrium include Monoclonal Gammopathy of Unknown Significance (MGUS), and plateau phase disease after therapy, implying that significant host-tumor interaction occurs. The clinical counterpart of escape is the progression from MGUS to MM or disease relapse after plateau phase (1).

Regulatory CD4^+^ T cells (Treg) and cytotoxic CD8^+^ T cells have emerged as the dominant effectors in host myeloma control in syngeneic transplantable murine myeloma models (2, 3) underpinning autologous graft versus myeloma as an important process in disease control (4). The dramatic success of Chimeric Antigen Receptor (CAR) T cell therapies in myeloma reinforces the vulnerability of the malignant plasma cell to T cell-mediated cytotoxicity, even though the durability of response remains an issue (5, 6). Changes in the BM microenvironment and endosteal niche are also crucial to malignant plasma cell dormancy and growth permissiveness. These changes involve not only Tregs and cytotoxic CD8^+^ T cells, but also NK cells, myeloid-derived suppressor cells (MDSC), and cytokine fluxes which promote inflammation and angiogenesis resulting in impaired tumor immunity and a favorable environment for tumor growth (7).

The clinical finding of an association between improved clinical outcome and reduced Treg/Th17 ratios (8) or Treg frequency (9, 10) and oligoclonal expansion of terminal effector CD8^+^ T cells (T_TE_) (11) suggests Treg and oligoclonal expansion of T_TE_ as key players of immune surveillance in MM. This further suggests that T cells may recognize myeloma antigens and differentiate into T_TE_ which are able to exert cytotoxicity against malignant plasma cells through perforin and granzyme expression. Until recently this possibility had not been demonstrated in an autologous setting, but the recent demonstration of cytotoxicity mediated by circulating oligoclonal expanded T_TE_ against autologous malignant plasma cells provides proof for this concept and an opportunity to elucidate potential myeloma antigens as well as harnessing these cells for therapy (12). However, the crucial factor, or gatekeeper, which allows for BM-residency of Treg and T_TE_, and therefore immune surveillance at the site of malignant disease is yet to be determined. It is also unknown whether BM-residency, per se, is necessary to confer tumor control since the spatial and temporal relationship between circulating and BM-resident T cells and myeloma cells and other cells of the BM microenvironment is not yet elucidated. We postulate however that BM-residency of immune cells can be considered as the “Green Card” which allows for permanent myeloma surveillance at the site of disease initiation and progression.

## Treg in Myeloma

The classical Treg is a subset of CD4^+^ T lymphocytes characterized by the surface CD25^+^CD127^low^ phenotype and expression of transcription factor forkhead box P3 (FoxP3) (13). If the homeostatic balance between Treg-mediated suppression and T effector cell activation is unbalanced in favor of effector activation, autoimmune disease results. In the case of malignancy, excessive Treg activity leads to suppression or exhaustion of effector cells and a lack of tumor immune surveillance. Compared to MGUS, in MM there is skewing of the Treg and pro-inflammatory Th17 cell balance in favor of Tregs, and this balance may be more important than absolute numbers of Treg cells (8). In our study of long-term survivors of myeloma, normal or reduced Treg/Th17 ratios were seen (14). Thus, although this is merely a correlative finding, it is possible that in patients who have active myeloma the immune system tips in favor of myeloma cell growth due to immunosuppressive mechanisms mediated by regulatory immune cells (15). This normal or reduced Treg/Th17 ratio, however, must be interpreted with caution as this ratio could be due to the increased number of Th17 cells which contribute to myeloma pathology by sustaining malignant plasma cell proliferation even from the early stage of disease (16–20) and contribute to osteoclastogenesis (21). Other studies emphasized the relationship between increased Treg frequency and adverse clinical features such as hypercalcemia and IgA myeloma subtype (10) or inferior clinical outcomes in myeloma patients (9, 10). The increased frequency of Treg reported in MGUS and MM patients suggests that Treg are important players but increased frequency does not predict evolution of MGUS to MM (22, 23).

The mechanism of Treg interactions with the myeloma microenvironment is unclear. It has been postulated that Treg are implicated in myeloma progression based on their contribution to the complex immunosuppressive environment *via* secretion of the cytokines interleukin (IL)-10 and transforming growth factor β (TGF-β) by APRIL/TACI dependent mechanisms (24), the CD39/CD73 adenosine pathway (25) and direct inhibition of effector T cell responses (26). Myeloma cells can also induce Treg expansion and activation by secreting type 1 interferon (2), or acquired Treg by transferring membrane proteins to Treg by trogocytosis (27, 28).

Polychromatic fluorescence-based flow cytometry, the benchmark technology to study T-cell diversity, has divided Treg based on CD25, CD45RA, and FoxP3 expression into three subsets; resting Treg (CD25^+^CD45RA^+^FoxP3^lo^), activated Treg (CD25^+^CD45RA^−^FoxP3^hi^) and non-suppressive Treg (CD25^+^CD45RA^−^FoxP3^lo^) (13). Expression of ectonucleotidase CD39 defines a subset of Tregs involved in the canonical CD39/CD73 adenosine pathway (29), a key immunosuppressive mechanism operating in tumor microenvironments (30, 31). Restrictions in the number of available fluorophores, and the complexity associated with spectral overlap, however, currently limits flow cytometry technology beyond 34 parameters (32). Mass cytometry time-of-flight (CyTOF) technology provides an alternative methodology, allowing for more than 50 different parameters without the limitations associated with spectral analysis and represents an important advantage in the field of T-cell biology (33). Recently by using the advantages afforded by mass cytometry and the unsupervised clustering flow self-organizing map (FlowSOM) algorithm, we interrogated the distribution of multiple subsets within the Treg compartment in the matched BM and peripheral blood (PB) samples of MGUS and Newly Diagnosed (ND)MM patients and demonstrated some novel findings (34).

In contrast to the established role of CD39^+^ Treg in suppressive tumor microenvironments (including colorectal, pancreatic, head, and neck cancer) (30, 31, 35), we found a trend toward increased CD39^−^ Treg within the Treg compartment in BM and PB of NDMM patients compared to MGUS patients (34). Treg expressing the ectonucleotidase CD39 in conjunction with CD73-expressing cells hydrolyze extracellular ATP and generate adenosine (29). Following the engagement with its cognate receptors extracellularly produced adenosine suppresses effector T-cell responses and induces MDSC (36). In humans, CD39^+^ Treg are defined as activated memory cells and are implicated in the suppression of Th17 responses and the control of autoimmunity (37). Thus, it may be speculated that although increased Tregs in myeloma may outweigh the number of myeloma permissive Th17 cells, the detrimental effect on Th17 cells may be compensated by reduced ability of Treg to suppress IL-17 production due to in increased proportion of CD39^-^ Treg.

In addition to CD39^−^ Treg frequency, their subset composition also distinguishes between MGUS and NDMM patients (34). The FlowSOM clustering algorithm identified two subsets that emerged within CD39^−^ Treg of NDMM patients which were negligible or absent in CD39^−^ Treg of MGUS patients. One subset was found in both BM and PB which phenotypically resembled activated Treg based on CD45RO, CD49d, and CD62L expression; another subset resembled BM-resident Treg based on CD69 expression, a key marker of tissue residency, and restricted location within the BM. Based on their low/lack of CD38 expression these two subsets of CD39^-^ Treg are likely resistant to anti-CD38 monoclonal antibody therapy and may limit its effectiveness in MM patients (38).

**Figure 1:**
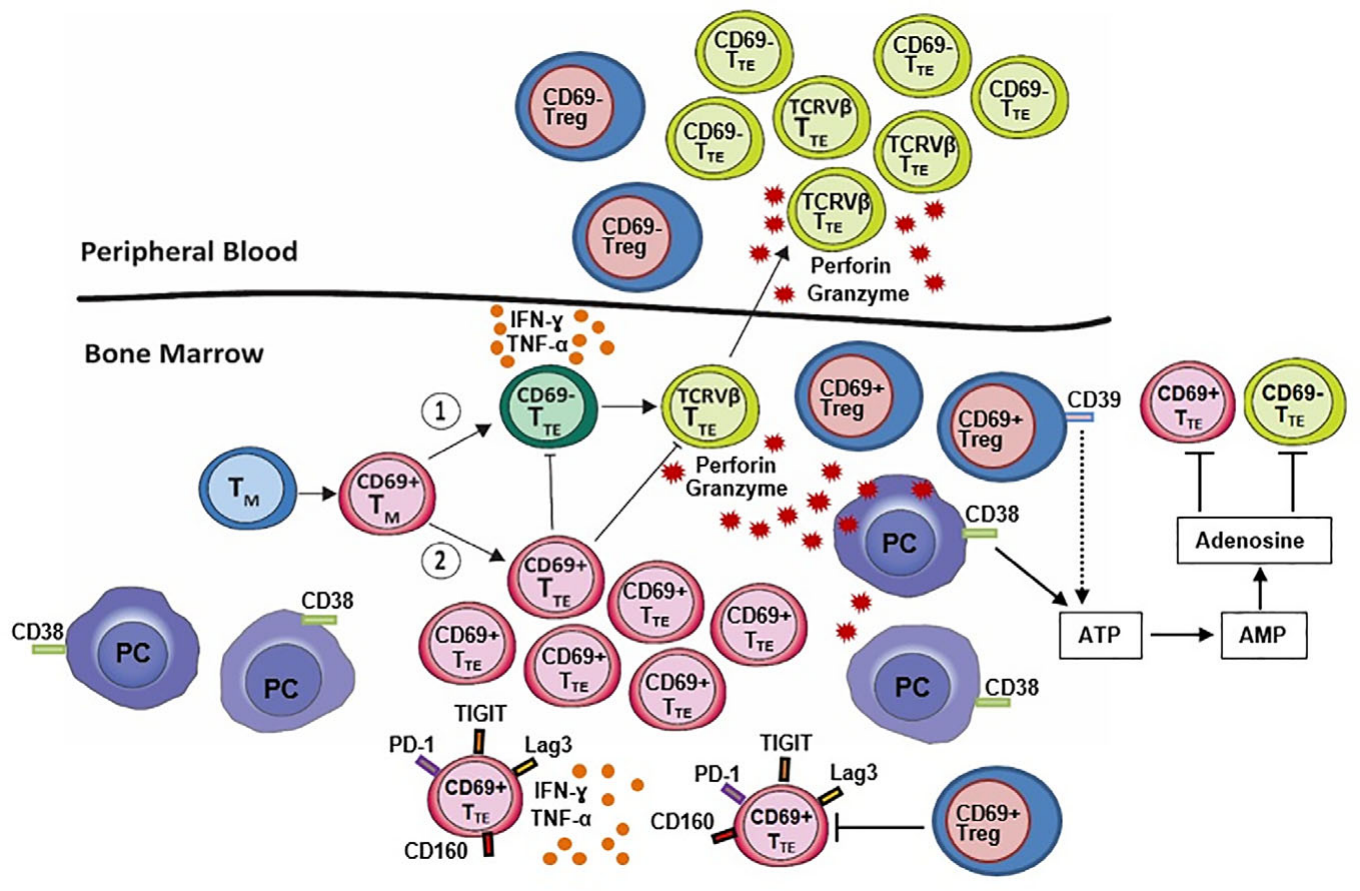
BM-resident CD69^+^ regulatory T cells (CD69^+^ Treg) decrease expression of CD39 which is required for the canonical CD39/CD73 adenosine pathway. This suggests that an alternative adenosine pathway involving ectoenzyme CD38, expressed on malignant plasma cells (PC), is active in the BM niches of NDMM patients. CD8^+^CD57^+^ terminal effector T cells (T_TE_) originate from memory T cells (T_M_) and may (1) undergo oligoclonal expansion into cytotoxic CD69^-^ T_TE_ (TCRVβ T_TE_) with high expression of perforin and granzyme B, the ability to eliminate autologous PC *in vitro* and circulate between BM and PB or, (2) remain within the BM as non-cytotoxic BM-resident CD69^+^ T_TE_ with high inhibitory checkpoint expression (PD-1, TIGIT, Lag3, CD160) possibly promoted by BM-resident CD69^+^ Treg. Both CD69^-^ T_TE_ and BM-resident CD69^+^ T_TE_ produce large amounts of the inflammatory cytokines interferon-γ (IFN-γ) and tumor necrosis factor α (TNF-α). CD69^−^ T_TE_ and CD69^+^ T_TE_ establish an inverse relationship within the BM-resident T cell compartment of NDMM patients. Changes in Treg and CD69^−^ T_TE_ and CD69^+^ T_TE_ within the BM-resident T cell compartment of NDMM patients may be critical for myeloma surveillance at the site of disease initiation and progression.

Our data provide additional evidence for divergence between Treg in elimination/equilibrium (MGUS) and escape stage (myeloma). This divergence has now been shown to be more than a skewing of balance between Treg and pro-inflammatory Th17 in favor of Treg, but also an active Treg differentiation process involving regulation of ectonucleotidase CD39 expression, activation and BM-residency (**Figure 1**). These changes in the Treg compartment have real potential to improve our understanding of the clinical stability in MGUS and disease progression into MM, and to further advance the clinical diagnosis, prognosis, and therapeutic implications for MM.

## Significance of Oligoclonal Expansion of CD8^+^T_TE_ Cells in Myeloma

In addition to Treg, oligoclonal expansion of CD8^+^CD57^+^ T_TE_ cells in myeloma patients have remained an attractive research topic and matter of debate for decades (11, 39–42). Although the clinical relevance of this phenomenon is well established, the biological explanation remain elusive, two mutually opposite concepts have been proposed. The first proposes that oligoclonal expanded T_TE_ represent exhausted and senescent T effector cells and are detrimental in myeloma immunity (39). The second proposes that oligoclonal expanded T_TE_ are key to disease control in myeloma as they are prognostically significant and correlate with improved survival (11).

Our recent research provides novel evidence supporting the idea that oligoclonal expanded T_TE_ are key players in myeloma disease control (12). We demonstrated that oligoclonal expansions not limited to PB CD8^+^ T_TE_ also occurred in CD8^+^ T_TE_ which belong to the pool of marrow infiltrating lymphocytes (MILs) (12). The mechanism underlining oligoclonal expansions of T_TE_ cells is unclear but infers reactivity against putative myeloma antigens. Such oligoclonal expansions of T_TE_ in MM patients may result from persistent stimulation of CD8^+^ T cells in the BM in the absence of effective clearance of the malignant clone and then oligoclonal expanded T_TE_ exit BM and circulate between BM and PB. Expanded oligoclonal CD8^+^ T_TE_ in BM and PB share same phenotype indicative of strong cytotoxic function with high expression of perforin and granzyme B and a Tbet^hi^Eomes^lo/neg^ signature (**Figure 1**). Low expression of the inhibitory checkpoints CD279 (PD-1), TIGIT, CD223 (Lag3) and CD160, suggests that these cells may not be exhausted and thus not an appropriate or optimal target for checkpoint blockade immunotherapy (12, 42, 43).

Significantly, oligoclonal expanded T_TE_ from the PB of NDMM patients are capable of eliminating autologous CD38^+^ plasma cells *in-vitro*, attributable to their perforin and granzyme B production (12). Direct testing of oligoclonal expanded T_TE_ residing in the BM for their myeloma reactivity remains challenging due to their limited numbers and small volume of diagnostic BM samples available for research. However, based on their cytotoxic function they likely resemble myeloma-reactive T cells reported in the BM of MM patients (44) and may be pivotal to favorable clinical outcome in MM patients receiving MILs as adoptive T-cell therapy (45). These cells also adopt a secretory phenotype and produce large amounts of the inflammatory cytokines interferon-γ (IFN-γ) and tumor necrosis factor α (TNF-α) (**Figure 1**) (12) aligning with the previously described senescence-associate secretory phenotype (SASP) (46–49).

These findings raise the possibility of the development of novel personalized therapies based on expanded clonal T_TE_ and a means of identifying new, previously occult, myeloma antigens.

## CD69: the Gatekeeper or “Green Card” Allowing BM-Residency of CD8^+^ T_TE_

It is likely that capacity of CD8^+^ T_TE_ to maintain immune surveillance over time at the site of malignant disease in MGUS and MM patients is linked to their retention within BM-resident T cell compartment, namely their BM-residency. Although understanding regulation of human BM-resident T cell compartment is in infancy, signals derived from endothelial cells (50) and attrition of memory stem-like TCF1/7^hi^ T cells (51) are considered important regulators factors in MGUS and MM patients.

Our recent investigations determined CD69 as a gatekeeper, or “green card”, allowing residence of CD8^+^ T_TE_ in the BM of MGUS and NDMM patients (12). CD69^+^ T_TE,_ being a constituent of MILs have many characteristics in common with resident memory T cells (T_RM_) including expression of CD69, a key marker of tissue-residency, cytokine production, and inhibitory receptor expression (**Figure 1**) (52). Only a minority of CD69^+^ T_TE_ express another marker of residency, CD103 (52). Non-circulating CD69^+^ T_RM_ lacking CD103 expression can be found in secondary lymphoid tissue following virus exposure and the expression of CD103 is a requirement for epithelial tissue-residency and adhesion to E-cadherin (53). CD69^+^ T_TE_ are not characterized by oligoclonal expansions, possess low perforin and granzyme expression and Tbet^lo^Eomes^hi^ transcriptional signature (12). Enhanced inhibitory receptor expression on BM-resident CD69^+^ T_TE_ marked them as exhausted and thus a suitable target for checkpoint inhibition immunotherapy.

BM-resident CD69^+^ T_TE_ are not restricted to myeloma, comprise approximately half of CD8^+^ T_TE_ in the BM of healthy controls, and a small proportion of CD8^+^ T_TE_ in the BM of MGUS patients. However, an inverse relationship between BM-resident CD69^+^ T_TE_ and their CD69^-^ T_TE_ counterpart encompassing oligoclonal expanded T_TE_ discriminates NDMM from MGUS patients and healthy controls (12). BM-resident CD69^+^ T_TE_ also maintain an inverse relationship with oligoclonal expanded CD69^-^ T_TE_ found in the PB of NDMM patients. The documentation of a unique relationship between BM-resident CD69^+^ T_TE_ and circulating cytotoxic CD8^+^ T_TE_ in myeloma is of great interest, as the cardinal feature of T_RM_ cells is a lack of equilibration with the circulating memory T-cell pool (54). It remains unclear whether CD69^+^ T_TE_ can be replenished from circulating CD69^-^ T cells or whether the self-renewal of CD69^+^ T is sufficient to maintain population stability.

Our data established that CD69^+^ T_TE_ independent of disease reside within the BM-resident T cell compartment, however, regulatory mechanism develop between CD69^+^ T_TE_ and CD69^-^ T_TE_ may impact immune surveillance in MGUS and NDMM patients.

## Discussion

In this mini-review, in the context of earlier published data, we discussed our recent data which suggest that the host-myeloma escape stage clinically defined by progression from MGUS to MM is characterized by lessening involvement of Treg in suppressive CD39/CD73 adenosine pathway and development of the regulatory mechanism between BM-resident CD69^+^ T_TE_ and their circulating CD69^-^ T_TE_ counterpart encompassing oligoclonal expanded T_TE_. We also discussed breaking discovery that clonally expanded T_TE_ are capable of eliminating autologous CD38^+^ plasma cells *in-vitro* and such play a key role in myeloma immunity.

It is surprising that Treg during myeloma escape decrease CD39 expression and become less involved in a key suppressive CD39/CD73 adenosine pathway operating in tumor microenvironment (30, 31, 35). Pertinent to their immunosuppressive fate, Treg must be able to adjust for this reduced involvement in the immunosuppressive pathway. Plausible adjustments are: reduced ability to suppress IL-17 production due to low CD39 expression and capacity of CD39^-^ Treg to produce IL-17 (37) a growth factor for myeloma (3). In particular BM-resident CD39^-^CD69^+^ Treg subset may be an important source of IL-17 at the site of disease initiation and progression. Lessening involvement of Treg in suppressive CD39/CD73 adenosine pathway is likely compensated by alternative adenosine pathway involving NAD(+)-consuming enzyme CD38 which is active in the BM niches of myeloma (55) and correlates with progression of myeloma (**Figure 1**) (56).

We discovered that CD69 acts as a gatekeeper, or “green card”, allowing residence of CD8^+^ T_TE_ in the BM (12). Our preliminary data suggest that the development of BM-resident CD69^+^ T_TE_ may be more closely related to the transition from T_M_ cells expressing CD69 rather than from CD69^+^ T_TE_ and that regulation of this transition may differ between NDMM and MGUS patients (12). Low perforin and granzyme expression by BM-resident CD69^+^ T_TE_ rule out their contribution to cytotoxic activity against myeloma cells. Cytotoxic activity against myeloma cells is limited to CD69^-^ T_TE_ encompassing oligoclonal expanded T_TE_ which circulate between BM and PB in NDMM patients (**Figure 1**). One of the possible functions of BM-resident CD69^+^ T_TE_ in myeloma is the recruitment of other immune cells from the circulation to the site of disease, as has been demonstrated with resident and circulating memory T cells in a murine vitiligo model (57). BM-resident CD69^+^ T_TE_ may potentially have a beneficial effect against myeloma by recruiting oligoclonal expanded cytotoxic CD69^-^ T_TE_ and maintaining dormancy of the malignant clone. Alternatively, the accumulation of CD69^+^ T_TE_ within MILs may contribute to local inflammation through the production of the pro-inflammatory cytokines IFN-γ and TNF-α and promote myeloma growth (58). Tracing BM-resident CD69^+^ T_TE_ within MILs and correlating their numbers with clinical outcome in MM patients receiving MILs as adoptive T-cell therapy (45) could provide essential insights into the role of BM-resident CD69^+^ T_TE_ in myeloma immunity.

It is possible that BM-resident CD69^+^ T_TE_ and circulating CD69^-^ T_TE_ cooperate in anti-tumor immunity based on the inverse relationship between CD69^+^ T_TE_ and CD69^-^ T_TE_ observed within the BM-resident T cell compartment in NDMM patients (12). We furthermore suggest that a competitive flux, or equilibrium, between BM-resident CD69^+^ T_TE_ and circulating CD69^-^ T_TE_ regulates the egress of myeloma-reactive CD69^-^ T_TE_ from the BM to PB and contributes to senescence (CD69^-^PD-1^lo^ T_TE_) or exhaustion (CD69^+^PD-1^hi^ T_TE_) of CD8^+^ T_TE_. This is also applies to the effectiveness of CAR T cells which are likely impacted by the ability to CAR T cells to reach disease sites and to avoid the development of senescence and exhaustion while remaining in the BM.

Further studies are required to determine the role of BM-resident cells including CD69^+^ T_TE_ and Treg, and circulating oligoclonal expanded CD69^-^ T_TE_ in the healthy and disease-affected BM microenvironments. These will require further functional studies of these subsets and their fluxes in the evolution of myeloma activity.

## Author Contributions

DJ wrote the manuscript with assistance from SV and JF and was reviewed by all other authors. All authors contributed to the article and approved the submitted version.

## Funding

This work and open access publication fees were funded by Sydney Blood Cancer Research and The Institute of Haematology, Royal Prince Alfred Hospital.

## Conflict of Interest

The authors declare that the research was conducted in the absence of any commercial or financial relationships that could be construed as a potential conflict of interest. 
